# Perceptual Richness of Retrieval Cues Enhances Memory for Emotional and Neutral Natural Scenes

**DOI:** 10.1111/psyp.70316

**Published:** 2026-05-15

**Authors:** Vera Ferrari, Andrea De Cesarei, Giorgio Li Pira, Mariagrazia De Gioia, Maurizio Codispoti

**Affiliations:** ^1^ Department of Medicine and Surgery University of Parma Parma Italy; ^2^ Department of Psychology University of Bologna Bologna Italy

## Abstract

Natural scenes are typically well‐categorized in meaning and efficiently recognized, even under perceptually degraded conditions. The present study investigated whether the perceptual quality of a retrieval cue influences the long‐term reconsolidation of visual memory. Participants were initially exposed to a series of images varying in emotional content. After a short delay, they completed an intermediate recognition memory test in which previously seen (“old”) and novel (“new”) images were presented in either a blurred or intact format. One week later, participants performed a final recognition task involving only intact old images—some of which had been tested previously (as blurred or intact), and others not tested at all. Recognition performance was generally enhanced for old pictures that had been previously tested, relative to non‐tested pictures, with a greater benefit observed for images tested in intact form. Although emotional images were overall better remembered than neutral images, the benefit of testing with intact cues was more pronounced for neutral than for emotional pictures. Event‐related potentials during the final task revealed a larger late positive component (LPC) for correctly recognized old versus new images, regardless of prior testing or emotional content. These findings suggest that recognition memory for natural scenes—while already robust—can be further strengthened through a single re‐presentation within an active retrieval context. Critically, the perceptual quality of the retrieval cue plays a key role, likely by promoting continued visual processing. Implications are discussed in relation to desirable difficulty and global/local processing theories in scene perception and memory.

## Impact Statement

This study reveals that the perceptual quality of retrieval cues critically influences long‐term memory reconsolidation for natural scenes, with intact images enhancing memory more than blurred ones. By combining behavioral and ERP evidence, it shows that detailed visual processing during active retrieval strengthens memory traces—especially for neutral pictures, reducing their typical memory disadvantage compared to emotional images. These findings challenge the desirable difficulty framework in visual memory, advancing our understanding of how perceptual and emotional factors interact to shape memory consolidation.

## 1 Introduction

Visual long‐term memory (vLTM) has been described as a large‐capacity system, especially when visually complex and semantically diverse stimuli such as natural scenes are used (Brady et al. 2008; Evans and Baddeley 2018; Konkle et al. 2010; Standing 1973). Several studies have demonstrated that vLTM can store a significant amount of detailed information, sufficient to distinguish a rich scene—not only an individual object—from many other exemplars within the same category (Hollingworth 2005; Konkle et al. 2010). Considering the inherently high memorability of natural visual scenes, it remains unclear to what extent this type of memory can still be improved. In the present study, we investigated how retrieval practice modulates memory consolidation of emotional and neutral natural scenes.

Recent research has investigated how top‐down manipulations during encoding may affect long‐term memory for natural scenes. Baddeley and Hitch (2017) compared different types of verbal and visual materials in terms of deep processing manipulations, which have played a central role in the study of episodic memory for individual words. According to the Levels of Processing (LOP) framework (Craik and Lockhart 1972), the depth of cognitive processing determines the durability of memory traces, with deeper semantic processing leading to better retention than shallow, surface‐level processing. In their study, Baddeley and Hitch manipulated the depth of processing using pleasantness judgments to induce deep encoding and found that visual stimuli—such as images of objects or natural scenes—exhibited a reduced LOP effect compared to words. This attenuated effect for visual material has been interpreted as stemming from the fact that visual stimuli comprise a rich array of features that are encoded rapidly and in parallel (Oliva and Torralba 2006; Potter et al. 2014), leading to automatic categorization at a basic level (Rosch 1978) or in terms of global scene properties (Oliva and Torralba 2001), even in the absence of explicit task instructions. In contrast, words can be encoded through a wider range of strategies, depending on the participant's adopted encoding approach (Craik and Tulving 1975). Extending these findings, Evans and Baddeley (2018) showed that memory consolidation for visual scenes, unlike verbal material, does not require an explicit intent to learn, as diagnostic cues for visual memory are more dependent on perceptual features and less on strategic semantic elaboration.

On the other hand, there is evidence that even simple multiple repetitions of the same picture exemplar during incidental encoding can produce a memory benefit in subsequent recognition tests (Ferrari et al. 2013). Moreover, when repetitions are distributed in time (i.e., the *spacing effect*), the memory enhancement is even larger compared to massed repetitions, and it remains evident even for pictures that are already highly memorable, such as emotional images. This behavioral pattern is supported by electrophysiological evidence showing that cortical correlates of memory strength—specifically, late centro‐parietal ERP positivities—increase in magnitude with the number of repetitions distributed across time (e.g., Carpenter et al. 2022; Ferrari et al. 2015; Megla and Woodman 2021). That repetitions, especially distributed ones, may enhance the probability of specific episodic retrieval is consistent with multiple‐trace theories of repetition (e.g., Bradley et al. 2015; Cepeda et al. 2006; Glenberg 1979; Hintzman 1988; Lansdale and Baguley 2008), which propose that repeating items during encoding results in multiple, separable episodic traces that facilitate the retrieval process.

Beyond manipulations during encoding, memory research has also extensively investigated the effects of retrieval itself on long‐term memory consolidation. Retrieval practice is considered “one of the most effective ways of solidifying new knowledge” (Bjork and Bjork 1992; McDermott 2021; Pyc and Rawson 2009; Roediger 3rd and Karpicke 2006b). This enhancement is generally observed in testing contexts, where participants take an intermediate test on previously encoded material. Crucially, the benefit persists even when the retrieval condition, cued only partially, is compared with a restudy condition in which participants re‐experience the entire previously learned material (Carrier and Pashler 1992; McDermott 2021; Roediger 3rd and Karpicke 2006a). This suggests that the improvement arises from the act of retrieval itself rather than from mere re‐exposure to the original stimulus. However, as with other memory mechanisms, this phenomenon has been investigated predominantly with verbal material—such as prose passages, scientific texts, word lists, and paired‐associate materials like foreign‐language translations (see Dunlosky et al. 2013)—with far less known about its applicability to visual material such as natural scenes.

The present study investigates whether retrieval practice can enhance memory for natural visual scenes. Moreover, considering that in real‐life situations the cues that trigger our memories rarely appear under the same perceptual conditions—due to changes in viewpoint, occlusion, illumination, or contextual factors such as exposure time and viewing distance (e.g., Kent et al. 2018; Loftus and Harley 2005; Masarwa et al. 2022; Wolfe and Kuzmova 2011)—it remains unclear how such perceptual changes at retrieval influence reconsolidation and subsequent memory. Thus, in the present study, we specifically examined the retrieval practice effect as a function of stimulus degradation during the testing phase. Participants were initially exposed to a series of images of natural scenes and, after a short interval, an intermediate recognition memory test (referred also as testing phase) was introduced with old images intermixed with previously unseen images. In the testing phase, both old and new images could be presented degraded (blurred) or intact. After 1 week, the same participants underwent a final recognition memory task on intact old pictures previously tested as blurred, intact, or never tested before (only seen in the initial encoding phase). The goal here is to explore whether, and to what extent, the retrieval process in the testing phase interacts with the quality of the cue in reconsolidating the new memory trace. If the reconsolidation process benefits of a further encoding of all the information that is present in the visual stimulus (i.e., both global shape and local details), then we expect a memory enhancement for intact, compared to blurred, cues. A different scenario could be predicted by Bjork's desirable difficulty hypothesis (Bjork 1994), which states that the introduction of difficulty (in this case, perceptual disfluency of the blurred scenes; Diemand‐Yauman et al. 2011) during the intermediate testing should improve final recognition performance (Alter et al. 2007); according to this, we may expect an advantage in long‐term memory for those images tested with a blurred cue, relative to the intact condition. Finally, if what matters is the recovery attempt itself that strengthens the memory trace (see the two‐stage framework of Kornell et al. 2015), both types of scenes (i.e., blurred and intact) can be equally effective in promoting reactivation of the original memory trace, since it is known that mnemonic performance (old‐new decision) can be based on a small amount of information and does not necessarily require the identification of detailed objects in the scene (Torralba 2009; Wolfe and Kuzmova 2011). If so, then we might expect similar reconsolidation for the blurred and intact images presented in the testing phase.

In the present study, pictures varied also in terms of emotional content, with the goal to explore whether the emotional memory enhancement, generally found in incidental encoding contexts (Bradley et al. 1992; Dolcos et al. 2017), would also be influenced by an explicit retrieval process. According to one hypothesis (Bradley et al. 2015; Ferrari et al. 2013, 2015), emotional images—both pleasant and unpleasant—tend to be remembered better over time because they trigger spontaneous retrieval processes that gradually strengthen memory traces. In support of this view, Ferrari et al. (2013), found that multiple repetitions in encoding produced a larger memory benefit for neutral, compared to emotional pictures, such that the general memory advantage for emotional cues was only evident in the most degraded condition (i.e., single presentation). Based on these findings, we can expect that the introduction of explicit retrieval phase may be more beneficial for neutral than for emotional images, particularly when these images are presented in their intact form.

Because recognition decisions may rely on both recollection and familiarity—processes that can be influenced by decision criteria (Donaldson 1996)—we concurrently recorded ERP activity during the final recognition task, providing a complementary measure to behavioral recognition performance. Specifically, the centro‐parietal late positivity, which is typically larger for correctly recognized old items than for new ones, has been proposed as a neural marker of memory strength (Curran and Doyle 2011; Ferrari et al. 2013; Voss and Paller 2008; Weymar et al. 2009; Wilding et al. 1995; Wilding and Rugg 1996).

## 2 Methods

### 2.1 Participants

A total of 25 university students (13 females; mean age = 22.9 years, SD = 2.5) from the University of Parma volunteered to participate in the experiment and provided written informed consent prior to participation. At the time of data collection, formal approval by an institutional ethics committee was not required under the applicable institutional regulations, as the ethics committee for non‐clinical human research (REB) had not yet been established. The experimental protocol conformed to the principles of the Declaration of Helsinki. The sample size was determined based on previous studies investigating memory performance and event‐related potentials (ERPs) (e.g., Ferrari et al. 2015, 2020; Weymar et al. 2011; Wirkner et al. 2013) and was further confirmed by an a priori power analysis (α = 0.05, power = 0.80), targeting a large effect size (partial η^2^ = 0.14; Cohen 1988). All participants had normal or corrected‐to‐normal vision, and none of them reported current or past neurological or psychopathological problems.

### 2.2 Materials

A total of 480 color photographs were selected, including images from the International Affective Picture System (IAPS; Lang et al. 2008)^1^ and additional pictures sourced from the Internet. The latter were chosen to match the semantic categories represented in the IAPS images.^2^ Half of the images depicted emotional content and half neutral content. Emotional stimuli were evenly divided into pleasant and unpleasant categories. Pleasant categories included erotic couples, romance, families with babies, puppies, and sports, whereas unpleasant categories included mutilations, human threat, animal threat, disgust, and illness. Neutral categories included people, urban and natural landscapes, and objects.

Pictures were selected such that two sets of 180 pictures included the same number of picture exemplars from each semantic category. One set of pictures was presented in the encoding phase and then as the old pictures in the final recognition task (1 week later), intermixed with the remaining 180 pictures that were not presented in the encoding phase (new pictures). The specific set of pictures serving as old or new was counterbalanced across participants. 120 additional pictures, equally distributed across categories were presented in the intermediate testing phase, serving as new pictures, intermixed with two‐thirds of the old pictures previously encoded. 14 buffer images were added, half to the beginning and half to the end of the encoding phase to avoid serial position effects and were not included in following phases.

### 2.3 Manipulation Parameters

In the encoding phase, pictures were presented as an animation, which lasted 8.7 s, and each frame in the animation represented the same picture in different degradation levels. Degradation was achieved by applying a low‐pass filter which blocked all spatial frequencies above an F0 value (expressed in cycles/image, cpi) and progressively increased passing spatial frequencies up to F0/3, below which all spatial frequencies were passed. At the beginning of the animation, the picture was presented for 2 s in the blurriest condition, and the F0 value was 22.62 cpi. Then, degradation was reduced in 20 steps of 100 ms which changed F0 by 0.175 cpi, until an F0 value of 256 cpi which corresponded to optimal viewing conditions was reached (see, e.g., De Cesarei and Codispoti 2011; De Cesarei and Loftus 2011). This intact image was shown for 1 s, after which the opposite degradation sequence was applied, and F0 was decreased in 20 steps from 256 to 22.62 in steps of 0.175, which lasted 100 ms each. The final blurred version was again shown for 2 s.

In the testing phase, static pictures serving as old or new in the old/new decision task were presented intact or blurred (low‐passed with a 256 or 22.62 cpi threshold) in two separate blocks. The order of blocks was counterbalanced across subjects.

Stimuli were displayed on a gray background of a 16‐in. monitor at 800 × 600 resolution and at a refresh rate of 120 Hz. The presentation of the gif in the encoding phase was managed by OpenSesame software (Mathôt et al. 2012). Stimulus presentation and data collection in the testing phase as well as the final recognition task were performed using E‐Prime software (Schneider et al. 2012). Pictures were viewed from 1 m and subtended a visual angle of 20.96° (horizontal) × 15.66° (vertical). A chinrest ensured that the distance remained constant within and across participants.

### 2.4 Procedure

**FIGURE 1:**
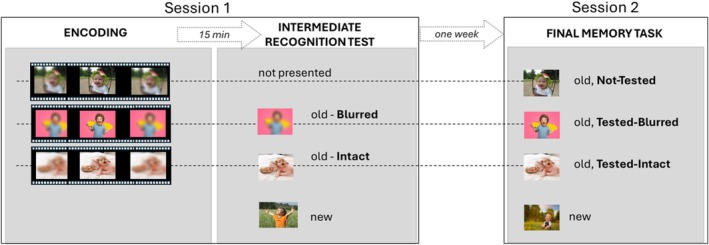
Procedure of the experimental sessions.

Figure 1 illustrates the schematic diagram showing the sequence of events in the present study. The experiment took place in two sessions, 1 week apart: the encoding phase followed by the intermediate test (testing phase) in session 1 and the final recognition memory task in session 2. Each session lasted about one hour and a half. At the end of session 2, participants were debriefed regarding the aims of the experiment.

After arrival at the laboratory, participants signed an informed consent form and filled in a brief fear questionnaire (the FSS III, Fear Survey Schedule; Wolpe and Lang 1964), also showing a few exemplars of the most arousing categories, to exclude people particularly sensitive to blood cues. Then the participant was accompanied to the experimental room, and written instructions were explained orally before the experiment began. In the encoding phase, participants were told that a series of gif stimuli would be presented (*n* = 180 plus 14 buffer stimuli) and that each of them should be viewed the entire time it was on the screen (8.7 s). A short 1‐min break was introduced halfway through the block. No mention was made about the upcoming recognition test (incidental encoding). Between the encoding and the testing phase, participants were engaged in a brief distractor task in which they filled few questionnaires regarding imagery skills (Questionario di Strategie Visive‐Verbali (QSVV), Antonietti and Giorgetti 1993; Verbalizer‐Visualizer Questionnaire (VVQ), Richardson 1977) that lasted about 15 min.

In the testing phase, participants were instructed to press one button if the picture stimulus had been seen before and another if it had not. Two blocks of 120 pictures each (60 old and 60 new, randomly intermixed and equally balanced in terms of emotional content) were presented. In one block, all pictures appeared in a blurred version, while in the other, all were intact. The order of the two blocks was counterbalanced across subjects. Each picture was presented for 1000 msec and followed by an intertrial interval (ITI) of 1500–1800 msec.

After 1 week, subjects returned to the laboratory for the final memory task. Unlike session 1, the EEG sensor net was attached before starting the experiment. The recognition task (old‐new decision) was similar to the one performed in session 1, but here all pictures (180 old and 180 new) were presented intact. Each picture was shown for 1000 ms, followed by a question “have you seen this picture before?” that guided participants to respond only after image offset, and this was to prevent the motor potential from interfering with the slow wave ERP.

### 2.5 EEG Recording and Data Reduction

In session 2, during the final recognition memory task, electroencephalogram (EEG) was recorded at a sampling rate of 1000 Hz using a 59 channel ElectroCap connected to a SA Instrument CO (San Diego, CA) UF‐64/72BA amplifier and in‐house developed software, using Cz as online reference. Impedance of each sensor was kept below 10 kΩ. Eye movements were recorded at a sampling rate of 1000 Hz from two bipolar couples of electrodes, placed respectively 1 cm above and below the right eye and 1 cm left and right to the side of the eyes. Both EEG and ocular signal were on‐line filtered from 0.01 to 100 Hz. Off‐line analysis was performed using Emegs (Peyk et al. 2011). First, eye movements were subtracted from the EEG on a trial‐by‐trial basis, based on the data from the monopolar horizontal and vertical EOG, and using an automated implementation of the regressive procedure (Gratton et al. 1983; Schlögl et al. 2007). Then, raw data were low‐pass filtered at 30 Hz. ERP averages were computed with a 200 ms baseline and a 1000 ms time window. Trials and sensors containing artifacts were detected through a statistical procedure (Junghöfer et al. 2000).

Specifically, the Emegs algorithm calculated the distributions of EEG amplitude, first derivative, and amplitude variability for each participant, trial, and sensor. Then, trial‐specific and sensor‐specific exclusion was carried out as described in Junghöfer et al. 2000, using a channel‐specific threshold of 0.25 (channel for standard exponent in goal function) and a channel‐generic threshold of 5 standard deviations to exclude channels. Then, the procedure proceeded to calculate whether each trial should be excluded, or in which excluded sensors should be interpolated from the remaining ones. This is done based on a goal function, which computes goodness of approximation based both on the number and on the positioning of bad sensors within a trial; the more bad sensors and the worse their distribution, the worse the approximation will be. Here, the worst acceptable goodness of approximation was set to 0.2. For the remaining trials, sensors containing artifacts were replaced by interpolating the nearest good sensors.

Trials were accepted for analysis if they were both accurate in their response (i.e., old vs. new), and with a good signal quality. A minimum of 215 accepted epochs (60% of total trials) was required for this experiment. Across participants, a mean percentage of 73% accepted epochs was achieved.

Processed data were then transformed to an average reference and baseline corrected (200 ms before picture onset) before subject averaging and analysis.

### 2.6 Data Analysis

EEG analyses were performed only on accurate trials (hit and correct rejection) in the final recognition memory task, 1 week after the encoding phase. For each subject, trials were averaged separately for each Testing condition (four levels: New, Tested–Intact, Tested–Blurred, Not‐Tested) and Emotion (two levels: Emotional, Neutral), resulting in eight ERP averages per participant. The minimum and maximum number of accepted trials across participants and conditions were as follows: for emotional and neutral pictures in the “new” condition (66–90 and 68–89, respectively), in the “tested intact” condition (17–30 and 11–29), in the “tested blurred” condition (12–29 and 4–26), and in the “not‐tested” condition (7–23 and 2–19).

To identify the ERP components of interest, time windows were selected based on previous studies investigating old/new ERP effects with natural scenes (e.g., Versace et al. 2010; Weymar et al. 2009). Two time windows were considered: an early window (300–500 ms) and a late window (500–800 ms). We performed a permutation analysis (1000 permutations) for the factors old‐new Conditions, Emotion, and their interaction, followed up by a cluster‐based correction for multiple comparisons. This preliminary analysis did not reveal a consistent or spatially extended old/new effect in an early time window (300–500 ms). In the 500–800 ms time window a significant main effect of Condition over central sensors, as well as a significant main effect of Emotion in the same spatiotemporal cluster, were found. No significant Condition × Emotion interaction was observed in this time window. The scalp topography of this effect was consistent with prior findings, predominantly involving centro‐parietal regions (Ferrari et al. 2013, 2015). Independence between sensor selection and subsequent statistical testing was ensured by defining the sensor selection procedure a priori, based on the grand‐average ERP across old–new conditions and participants, and not on the testing condition, the emotional modulation, or their interaction, which constituted the main focus of the analyses. Therefore, this approach does not introduce circularity into the statistical analyses. Accordingly, only sensors showing a significant old/new difference in paired *t*‐tests (α = 0.01), computed collapsing across all testing conditions and emotional categories within this late time window were retained and included in the 2 × 4 ANOVA reported in the results section (selected sensor cluster: FC1, FCz, FC2, C3, C1, Cz, C2, C4, CP3, CP1, CPz, CP2, CP4). An initial analysis also investigated laterality differences between left and right centro‐parietal regions, but no significant differences were found. Therefore, the final analyses were averaged across hemispheres. Recognition accuracy was also analyzed in a repeated measure ANOVAs, with the two factors Testing condition (4) and Emotion (2). Greenhouse– Geisser corrections were applied where relevant. The partial eta squared statistic (η^2^_p_), indicating the proportion between the variance explained by one experimental factor and the total variance, has been calculated and reported.

## 3 Results

### 3.1 Recognition Accuracy

**FIGURE 2:**
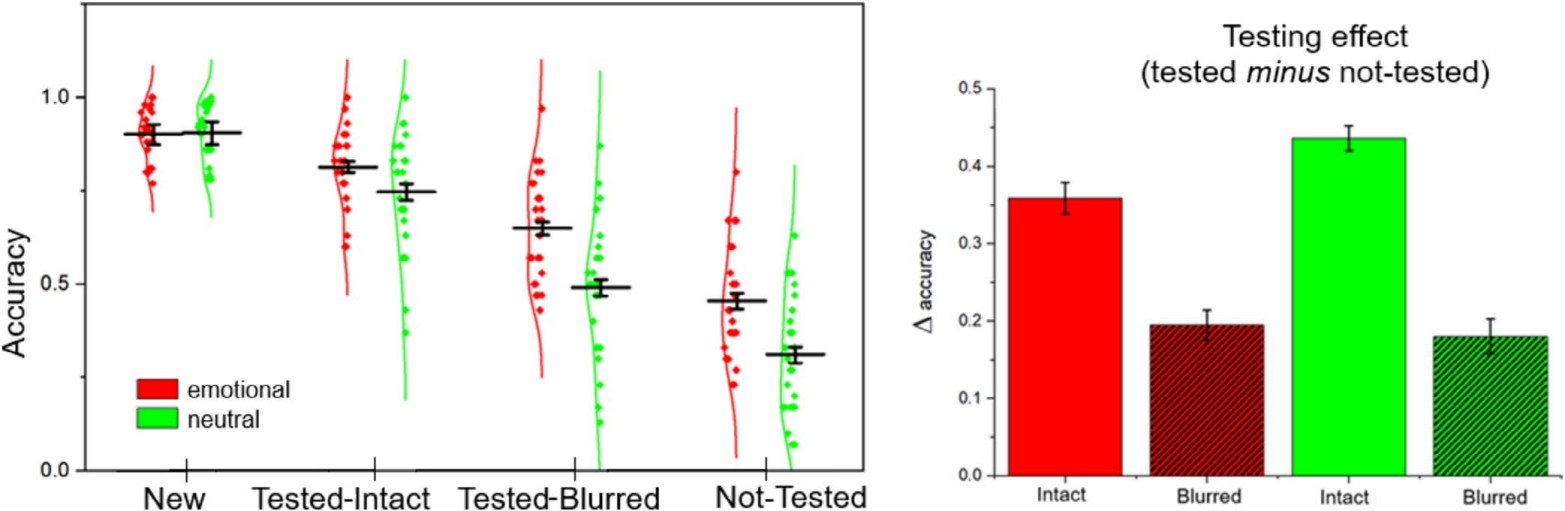
Memory performance 1 week after the initial encoding. On the left, recognition accuracy across conditions for emotional (red) and neutral (green) stimuli. Each dot represents a single participant. The distributions are illustrated using violin plots, which reflect the kernel density of the data. Horizontal black bars indicate the mean ± within‐participant standard error of the mean (SEM). Conditions include New, Tested‐Intact, Tested‐Blurred, and Not‐Tested, all relative to session 1. On the right, the bar graph shows the magnitude of the testing effect (tested—not tested) separately for emotional and neutral pictures, tested in their intact and blurred form. Error bars represent within‐participants standard errors of the mean (O'Brien and Cousineau 2014).

Figure 2 illustrates the recognition rate in the final memory task conducted 1 week after the initial encoding, including both the pictures presented during the testing phase (tested‐intact or tested‐blurred)—regardless of whether they were correctly recognized—and the pictures that were only seen during the initial encoding and not included in the testing phase (not‐tested). In this final task, old and new pictures were all intact. New pictures had never been presented before. In the overall analysis, significant effects were obtained as a function of Testing condition, F(3, 72) = 129.30, *p* < 0.001, η^2^_p_ = 0.843, and Emotion, F(1, 24) = 39.03, *p* < 0.001, η^2^_p_ = 0.619, and their interaction, F(3, 72) = 12.41, *p* < 0.001, η^2^_p_ = 0.341. Simple main effects tests indicated a significant effect of testing condition for both emotional, F(3, 72) = 91.16, *p* < 0.001, η^2^_p_ = 0.792, and neutral, F(3, 72) = 112.52, *p* < 0.001, η^2^_p_ = 0.824, pictures. In both cases, tested pictures, both intact and blurred, prompted a higher hit rate than not‐tested pictures [emotional: tested‐intact vs. not‐tested, F(1, 24) = 225.13, *p* < 0.001, η^2^_p_ = 0.904; tested‐blurred vs. not‐tested, F(1, 24) = 79.51, *p* < 0.001, η^2^_p_ = 0.768; neutral: tested intact vs. not‐tested, F(1, 24) = 401.24, *p* < 0.001, η^2^_p_ = 0.944; tested blurred vs. not‐tested, F(1, 24) = 53.02, *p* < 0.001, η^2^_p_ = 0.688]. Again, for both emotional and neutral pictures, intact condition always prompted a better performance compared to the blurred condition [emotional, F(1, 24) = 60.56, *p* < 0.001, η^2^_p_ = 0.716; neutral, F(1, 24) = 147.80, *p* < 0.001, η^2^_p_ = 0.860]. Recognition accuracy for new pictures was always higher than for old pictures of any kind [Fs(1, 24) > 8.60; ps < 0.01, η^2^_p_s > 0.264]. The interaction Testing × Emotion indicated that the recognition enhancement for emotional, compared to neutral, content was highly evident in the not‐tested and in the blurred condition, Fs(1, 24) > 27.35, ps < 0.001, η^2^_p_ > 0.533, but was significantly attenuated in the intact condition, F(1, 24) = 5.29, *p* = 0.031, η^2^_p_ = 0.181, compared to both the blurred, Emotion × Testing (2 levels: intact, blurred) F(1, 24) = 10.57, *p* = 0.003, η^2^_p_ = 0.306 and the not tested condition, Emotion × Testing (2 levels: intact, blurred) F(1, 24) = 7.97, *p* = 0.009, η^2^_p_ = 0.249. Hit rate for emotional and neutral pictures in the new condition did not differ, F(1, 24) = 0.13, *p* = 0.723, η^2^_p_ = 0.005.

### 3.2 Old‐New ERP Modulation (Window 500–800 ms)

**FIGURE 3:**
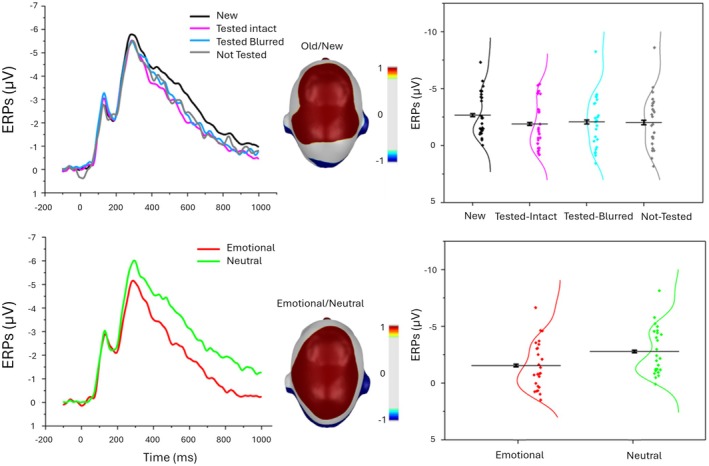
ERP results in the final memory task. ERP waveforms (sensor group) for the testing effect and the emotional modulation are reported (left). Scalp topography (top view) shows the t‐tests between old and new conditions (above) and emotional vs. neutral conditions (below) in the window 500–800 ms. On the right, the LPC in the four testing conditions (top right) and in the two emotional conditions (lower right) is reported. The distributions are illustrated using violin plots, which reflect the kernel density of the data. Horizontal black bars indicate the mean ± within‐participant standard error of the mean (SEM). Error bars represent within‐participant standard error of the mean.

Figure 3 illustrates the ERP results obtained in the final memory task. The sensor group involved in the discrimination between old and new pictures revealed a significant main effect of Testing condition, F(3, 72) = 4.37, *p* = 0.008, η^2^_p_ = 0.154, with a more positive ERP waveform for each correctly recognized old picture relative to new stimuli, regardless of the testing condition [new vs. tested‐intact, F(1, 24) = 23.13, *p* < 0.001, η^2^_p_ = 0.491; new vs. tested‐blurred, F(1, 24) = 8.23, *p* = 0.008, η^2^_*p*_ = 0.255; new vs. not‐tested, F(1, 24) = 6.45, *p* = 0.018, η^2^_p_ = 0.212]. Pairwise comparisons conducted to examine ERP differences among the different levels of the factor Old did not reveal any significant effects. This sensor group also showed a main effect of Emotion, F(1, 24) = 55.60, *p* < 0.001, η^2^_p_ = 0.699, indicating a larger positive wave for emotional compared to neutral pictures. However, there was no significant interaction between Testing and Emotion, F(3, 72) = 0.12, *p* = 0.865, η^2^_p_ = 0.005.

## 4 Discussion

In this study, we investigated how administering an old‐new recognition test following an initial incidental encoding phase influences the reconsolidation of memory for natural scenes, as a function of the perceptual quality of the retrieval cues. Memory for the images, assessed 1 week after initial encoding, was significantly enhanced for pictures that were re‐tested immediately after encoding, compared to those seen only during the initial phase (not‐tested condition). Critically, this reconsolidation effect was substantially stronger when the recognition test involved intact images, rather than blurred versions of the same scenes. While emotional pictures generally elicited stronger recognition memory than neutral ones, the benefit of repeated exposure to intact images was more pronounced for neutral stimuli. Furthermore, ERP analyses revealed greater centro‐parietal positivity during the final memory task for stimuli correctly identified as old, regardless of prior testing condition (tested‐intact, tested‐blurred, or not‐tested). This neural marker was also modulated by the emotional content of the images; however, the effects of emotionality and old‐new discrimination appeared to operate independently.

Memory research using verbal material has long emphasized the importance of active retrieval of previously encoded items, with retrieval effort playing a critical role in long‐term retention. Specifically, the more effortful the retrieval attempt, the greater the long‐term memory benefit—a principle known as the *desirable difficulty* account (Bjork 1975, 1994). Supporting this, studies have shown that presenting stimuli in degraded formats—such as fonts that are difficult to read or words in very small print—can induce subjective experience of disfluency, prompting individuals to engage in deeper processing strategies that enhance long‐term memory (Diemand‐Yauman et al. 2011).

The present study extends this line of research to the visual domain by examining the effects of perceptual disfluency in the retrieval of complex natural scenes. Specifically, images were degraded using Gaussian blurring, calibrated to preserve the overall gist of the scenes, in line with previous work (De Cesarei and Codispoti 2008, 2011; De Cesarei and Loftus 2011). Prior studies have shown that such stimuli support high levels of old–new recognition accuracy (Kent et al. 2018; Wolfe and Kuzmova 2011). Due to technical issues, we were unable to collect behavioral data during the intermediate memory phase. However, a pilot study using the same stimuli, tested with the same paradigm, indicated that hit rates for intact and blurred old pictures were very similar, suggesting that the perceptual information provided by the blurred images was sufficient to access the underlying memory trace. However, for new images, the false alarm rate was slightly higher in the blurred condition, possibly reflecting a shift in response criterion triggered by the perceptual difficulty (i.e., Kent et al. 2018)—consistent with the intended effect of our experimental manipulation. The main behavioral findings show that blurred images were less well remembered at long‐term testing, which may reflect differences in how these cues engaged retrieval‐related processes during the testing phase. In light of the pilot data, this pattern appears to be consistent with the hypothesis that perceptual disfluency may influence reconsolidation processes—not in the expected direction of strengthening memory, but potentially by weakening it relative to the reconsolidation prompted by intact images. Notably, intact visual cues were associated with better memory performance, suggesting that for complex natural scenes, factors beyond retrieval effort—particularly those related to perceptual processing—may play a more substantial role in shaping memory outcomes.

It is important to note that prior research on perceptual disfluency and memory has primarily focused on written verbal stimuli (e.g., Yue et al. 2013). While identification of characters/digits relies on the discrimination of local features (e.g., differentiating between similar characters such as C and G), natural scenes contain a broader range of information (De Cesarei et al. 2017), encompassing both global elements (e.g., sky, buildings) and fine‐grained local details (e.g., facial features). One possibility is that, contrary to the desirable difficulty hypothesis that applies to simpler stimuli such as characters and digits, recognition of natural scenes benefits rather than suffers from richer visual information at retrieval, as it allows for a stronger memory consolidation.

The present results suggest that global information—available in both blurred and intact pictures—was sufficient to discriminate between old and new images during testing in Session 1. However, access to local information, which was only available in the intact pictures, further strengthened the reconsolidation process, making these images more easily recognizable in the long term. It is important to note, however, that even degraded cues contributed to memory consolidation, as evidenced by differences between the blurred and untested conditions. These findings support the hypothesis that two main cognitive processes are engaged during picture viewing: one is the active retrieval prompted by task instructions, and the other is incidental encoding that occurs spontaneously as a secondary process (Buckner et al. 2001). When the picture provides the most comprehensive information—as in the case of intact images containing both global and local features presented at testing—the resulting memory trace is more strongly consolidated.

One possible alternative explanation for the observed advantage of intact over blurred images in the final recognition memory task concerns the degree of perceptual similarity between experimental phases. Specifically, since all images were presented in high resolution during the final test, images that had also been presented intact during the intermediate recognition phase may have benefited from a closer perceptual match between study and test. This overlap could have enhanced recognition performance through mechanisms such as perceptual fluency or transfer‐appropriate processing, whereby memory retrieval is facilitated when the cognitive operations or perceptual features at test align with those engaged during encoding (Roediger 3rd et al. 2002). In contrast, images previously viewed in a blurred format may have suffered from a mismatch between their degraded intermediate presentation and the intact final test format, thereby reducing recognition accuracy. From this perspective, the observed memory advantage may not solely reflect differences in encoding or recognition strength, but rather the influence of perceptual congruence across phases on retrieval success. Using blurred images—rather than intact ones—in the final recognition task could reveal a different pattern of results. Specifically, the differences between images previously studied in blurred or intact form may be attenuated or even eliminated. Such an outcome would suggest that local information is stored and retrieved flexibly and can operate independently of global information. Depending on task demands or contextual cues, either level of information may support recognition (Brooks et al. 2010; Castelhano et al. 2019). Conversely, if the same pattern of results observed in the present study emerges regardless of the test format, it would imply that global and local information are tightly integrated within the memory trace, with both contributing to retrieval irrespective of the final cue type. This issue warrants further investigation in future research.

Importantly, the interaction between cue quality (intact vs. blurred) and emotional content challenges the idea that perceptual similarity alone accounts for the observed effects. Although both emotional and neutral images were similarly affected by perceptual congruence between the intermediate and final tests, the recognition advantage of intact cues was more pronounced for neutral images. This pattern suggests that neutral images may have elicited more detailed encoding of perceptual features during retrieval, thereby compensating for their typical disadvantage in emotional memory comparisons. These findings further support the interpretation that extensive visual processing occurs during the retrieval task. These findings align with prior work showing that neutral images can benefit from repetition‐based memory enhancement, particularly when re‐exposure occurs over time (Ferrari et al. 2013). Such improvements likely depend on visuo‐spatial perceptual details rather than semantic factors. In contrast, emotional images tend to elicit deeper and more automatic processing, which includes enhanced attention and visual exploration—especially under degraded or competitive conditions (e.g., blurred stimuli or non‐tested images).

Although it is highly unlikely that a single re‐exposure alone could produce such a marked improvement in memory, it raises the question of what specific role the retrieval task—namely, the “old‐new” discrimination—plays in driving the observed enhancement. Does the improvement stem from the explicit attempt to access the memory trace, or is it more generally due to the level at which the stimulus is processed, regardless of the retrieval demands? Future studies could address this hypothesis by comparing different tasks that vary the type of memory (e.g., episodic vs. semantic) involved in the intermediate testing phase and the level of processing required (Baddeley and Hitch 2017).

In this study, ERP components were analyzed to determine whether recognition memory performance was driven by multiple processes that could be disentangled using distinct neural signals. Overall recognition can reflect a combination of explicit and implicit memory processes, as well as perceptual fluency, which may enhance familiarity (Voss et al. 2012). After exploring whole‐scalp activity, the ERP analysis focused on the centro‐parietal late positive component (LPC; Curran and Doyle 2011; Finnigan et al. 2002), where old–new differences were most reliable in terms of amplitude and scalp topograhy extent. This aligns with previous recognition memory studies showing that LPC is modulated by both memory strength and the emotional content of natural scenes (e.g., Ferrari et al. 2013).

In the current study, the LPC was clearly enhanced for all correctly recognized old pictures relative to new ones, but did not differ across old items based on their prior testing history. Notably, we did not observe a larger LPC for previously tested items—even those presented in intact form—despite these conditions producing better behavioral recognition performance. This may seem surprising given that LPC amplitude often tracks behavioral performance in other contexts. For example, Rosburg et al. (2015) reported a larger left‐parietal old–new effect (500–900 ms) for previously tested words compared to untested ones.

A critical factor may be the long retention interval used in our study (1 week vs. within‐session test). Longer retention intervals are thought to promote the emergence of highly consolidated memories, whereas weaker traces typically decay rapidly within the first hour to first day after learning (Yonelinas and Levy 2002; Murre and Dros 2015). In the testing literature, benefits of prior testing over restudy become more pronounced with longer delays (Roediger 3rd and Karpicke 2006a, 2006b), and the spacing effect is similarly amplified under long retention intervals (Cepeda et al. 2006; Glenberg and Lehmann 1980). Because our ERP analyses included only correctly recognized pictures, it is plausible that the items successfully retrieved after 1 week were those with relatively strong memory traces, regardless of their prior testing condition. This could explain the similar LPC amplitudes observed across the different old‐item conditions. The complexity of the relationship between neural processing and the subjective experience underlying old/new decisions should not be overlooked. As previously noted (Rosburg et al. 2015; Voss et al. 2012), divergent patterns in behavioral and electrophysiological measures suggest that the recognition of natural scenes may be supported by multiple, potentially distinct mechanisms.

Taken together, these findings indicate that long‐term memory for natural scenes can be enhanced simply by re‐presenting the stimuli and asking participants to make a memory judgment. This active retrieval context appears to prompt deep visual exploration, allowing the encoding process to benefit from the richness of perceptual details, thereby further strengthening the memory trace. This finding aligns with previous evidence suggesting that natural scenes offer limited potential for additional semantic elaboration, as relevant diagnostic cues are more likely to rely on perceptual rather than semantic features (Evans and Baddeley 2018).

## Author Contributions

**Vera Ferrari:** conceptualization, writing – original draft, writing – review and editing, methodology, data curation, supervision, project administration, investigation. **Mariagrazia De Gioia:** investigation, data curation, writing – review and editing, formal analysis. **Maurizio Codispoti:** methodology, conceptualization, supervision, writing – original draft, project administration, funding acquisition, investigation, resources. **Andrea De Cesarei:** methodology, formal analysis, software, writing – review and editing, investigation. **Giorgio Li Pira:** investigation, data curation, formal analysis, software, visualization.

## Funding

The authors have nothing to report.

## Ethics Statement

This study was performed in line with the principles of the Declaration of Helsinki.

## Consent

Written informed consent was obtained from all individual participants included in the study.

## Conflicts of Interest

The authors declare no conflicts of interest.

## Supporting information


**Data S1:** Supporting Information.

## Data Availability

The data that support the findings of this study are openly available in OSF at https://osf.io/efh46/?view_only=d07f92d0ed554ad599a7a13ed7555223, reference number DOI https://doi.org/10.17605/OSF.IO/EFH46.

## Endnotes

^1^

^1^ The same experimental paradigm described in this manuscript was used in an online study (PsychoPy, Peirce et al. 2019) conducted on 14 subjects (unpublished data). The results in the final memory task (after 1 week from the initial encoding and testing) were very similar to those found in the present study (testing condition, F(2,26) = 72.90, *p* < 0.001, η^2^_*p*_ = 0.849; Emotion, F(1,13) = 24.27, *p* < 0.001, η^2^_*p*_ = 0.651, interaction, F(2,26) = 6.67, *p* < 0.01, η^2^_*p*_ = 0.339), demonstrating the reliability of the online method compared to the laboratory study. The online study also allowed us to collect behavioral data during the intermediate testing phase. Hit rates (old‐new decision) in the intermediate phase showed no significant difference between intact and blurred images (0.71 vs. 0.72, *p* > 0.05). False alarms, on the other hand, were higher for the blurred images (F(1,13) = 10.50, *p* < 0.01, η^2^_*p*_ = 0.447) indicating a shift toward a more liberal response bias for the blurred, compared to the intact, images.

^2^
IAPS numbers: pleasant, 1605, 1630, 1659, 2151, 2152, 2332, 2342, 2345, 2347, 2351, 3262, 4597, 4599, 4610, 4611, 4614, 4617, 4641, 4647, 4658, 4659, 4660, 4687, 4693, 4694, 4800, 8031; 1460, 1595, 1710, 2060, 2075, 2314, 2340, 2360, 2655, 4604, 4616, 4624, 4626, 4640, 4645, 4653, 4666, 4668, 4676, 4680, 4690, 4692, 4697, 4698, 8179, 8370, 8499; unpleasant, 1111, 1120, 1205, 1300, 1304, 1930, 2717, 3000, 3001, 3015, 3140, 3170, 3195, 3212, 3261, 3350, 3400, 6212, 6230, 6570, 6821, 6834, 9120, 9301, 9326, 9412, 9909, 1052, 1302, 1303, 1525, 1931, 2703, 2730, 3053, 3064, 3068, 3069, 3080, 3100, 3150, 3213, 3500, 6242, 6350, 6838, 9183, 9322, 9410, 9414, 9425, 9594, 9905, 9940; neutral, 2050, 2102, 2190, 2273, 2372, 2374, 2393, 2394, 2396, 2397, 2493, 2506, 2512, 2515, 2560, 2593, 2745, 5455, 5471, 5500, 5531, 5635, 5875, 5900, 7001, 7026, 7036, 7041, 7057, 7495, 7550, 7595, 7632, 2039, 2217, 2221, 2305, 2370, 2382, 2383, 2390, 2410, 2435, 2500, 2501, 2513,2575, 2579, 2594, 2595, 5130, 5390, 5410, 6000, 7011, 7030, 7032, 7033, 7050, 7058, 7061, 7100, 7165, 7493, 7513, 7560, 7620, 7950. These IAPS pictures were complemented by other pictures of similar affective content and scene composition. This expansion was carried out by ensuring that the additional pictures were similar in content to those selected in Ferrari et al. (2013).
