# Supplementary material for: Perceptual Richness of Retrieval Cues Enhances Memory for Emotional and Neutral Natural Scenes
Source: Psychophysiology. 2026 May 15;63:e70316. doi: 10.1111/psyp.70316 (PMC13178402; doi:10.1111/psyp.70316)
Supplement: Supplementary file 1 — Data S1: Supporting Information. [file PSYP-63-e70316-s001.docx]

Statement

This study reveals that the perceptual quality of retrieval cues critically influences long-term memory reconsolidation for natural scenes, with intact images enhancing memory more than blurred ones. By combining behavioral and ERP evidence, it shows that detailed visual processing during active retrieval strengthens memory traces—especially for neutral pictures, reducing their typical memory disadvantage compared to emotional images. These findings challenge the desirable difficulty framework in visual memory, advancing our understanding of how perceptual and emotional factors interact to shape memory consolidation.
